# A Systematic Review of Human Papillomavirus Vaccination Challenges and Strategies to Enhance Uptake

**DOI:** 10.3390/vaccines12070746

**Published:** 2024-07-06

**Authors:** Mansour Tobaiqy, Katie MacLure

**Affiliations:** 1 Department of Pharmacology, College of Medicine, University of Jeddah, Jeddah P.O. Box 45311, Saudi Arabia; 2 NHS Scotland, Aberdeen AB10 1AB, UK

**Keywords:** human papillomavirus, vaccination, challenges, strategies, vaccine uptake, HPV vaccine programs, evidence-based interventions

## Abstract

Human papillomavirus (HPV) vaccination has revolutionized cervical cancer prevention. Clinical trials confirm that the quadrivalent (HPV types 6, 11, 16, 18) and bivalent (HPV types 16, 18) vaccines effectively prevent HPV infections and cervical neoplasia. The latest HPV vaccine protects against nine virus types responsible for 90% of cervical cancer cases globally. Despite their undoubted effectiveness in reducing morbidity and mortality associated with HPV infections, challenges in vaccine coverage and uptake persist. The current study aimed to identify the primary challenges associated with HPV vaccination, propose effective strategies to improve vaccination uptake, and compile relevant evidence into a comprehensive overview to inform policy and practice. A systematic review protocol, following PRISMA-P and PRISMA guidelines, was established. Articles were sourced from the Web of Science using keywords from a comprehensive review of HPV vaccination challenges and strategies. Studies published between 1 January 2020, and 1 May 2024, including RCTs and observational, qualitative, and cross-sectional studies, were included, while reviews, protocols, and commentaries were excluded. Titles, abstracts, and full texts were screened per PRISMA guidelines. The review identified five key strategies to improve HPV vaccination uptake: parental and school engagement, use of technology and multimedia tools, healthcare providers’ role, multicomponent interventions, and targeted interventions for immigrant groups. This review emphasized the need for a multifaceted approach to improving vaccination rates, offering a robust foundation for policy and stakeholder initiatives.

## 1. Background

Over the past twenty years, significant progress has been made in the development and efficacy of human papillomavirus (HPV) vaccines alongside a marked increase in global vaccination initiatives. Several countries have accumulated valuable experience in delivering HPV vaccines to adolescent girls through pilot programs, demonstration projects, and nationwide implementations, most of which occurred in the four years preceding 2020 [1,2].

HPV is a highly contagious virus linked to cervical cancer, posing a significant public health challenge both domestically and worldwide. The introduction of HPV vaccines has significantly improved the prevention of cervical cancer and other HPV-related morbidity and mortality [2,3,4].

A recent study in England evaluated the national HPV vaccination program, initiated in 2008 for girls aged 12–13, and included additional efforts for older teenagers. Results revealed that vaccinated women had an 83.9% reduction in cervical cancer and a 94.3% reduction in grade three cervical intraepithelial neoplasia (CIN3) rates compared to those unvaccinated [5].

Of note, a considerable number of clinical trials have shown that the quadrivalent vaccine (targeting HPV types 6, 11, 16, and 18) and the bivalent vaccine (targeting HPV types 16 and 18) are highly effective in preventing persistent HPV infections and CIN, which are critical precursors to cervical cancer. These vaccines have been approved and are now available on the market, demonstrating sustained immunity and strong antibody responses, which suggest long-term protection. Additionally, research indicates that the bivalent vaccine offers significant cross-protection against other HPV types [6,7,8,9,10]. Gardasil 9, the latest and most comprehensive HPV vaccine, protects against nine HPV types: 6, 11, 16, 18, 31, 33, 45, 52, and 58. These types are responsible for approximately 90% of cervical cancer cases globally [11].

Implementing three-dose vaccine campaigns in young girls has posed challenges, including difficulties in ensuring adherence to multiple dosing regimens in some ethnic groups, increased costs, and unavailability of health insurance [12]. This has prompted consideration for transitioning to two or even one dose to enhance vaccination efforts [13,14]. Notably, administering a single dose of the HPV vaccine can potentially enhance vaccine uptake [12,13]. Evidence indicates that a single-dose routine vaccination could prevent nearly as many cervical cancers as the two-dose regimen if the vaccine’s protective duration exceeds 20 to 30 years. This approach would be more efficient, easier to implement, and less costly [12,13,14]. Furthermore, during the 18-month trial period, single-dose bivalent and nonavalent HPV vaccines demonstrated high effectiveness in preventing new persistent oncogenic HPV infections, comparable to the protection offered by multidose regimens [15].

In 2020, the World Health Organization (WHO) launched the Global Cervical Cancer Elimination Initiative to eradicate cervical cancer [16]. This initiative emphasizes prevention, screening, and treatment. By 2030, it aims to reduce the global incidence of cervical cancer to fewer than four cases per 100,000 women. Key targets include vaccinating 90% of girls by age 15, screening 70% of women with high-performance tests at least twice by age 45, and ensuring 90% of identified cases receive treatment [16]. However, the feasibility of such a comprehensive approach across diverse socio-economic and healthcare settings globally needs to be adequately addressed [17,18,19]. Moreover, the initiative’s reliance on achieving high vaccination rates, widespread screening, and ensuring timely treatment overlooks women’s numerous barriers to healthcare access in many regions, including financial constraints, cultural stigmas, and infrastructure deficiencies [17,18,19]. Additionally, the target of reducing cervical cancer incidence to fewer than four cases per hundred thousand women per year by 2030 seems overly optimistic, given the current disparities in healthcare systems and resource allocation worldwide [15].

### 1.1. Safety Aspects Related to HPV Vaccination

While initial safety evaluations were conducted during efficacy trials, regulatory bodies such as the European Medicines Agency (EMA) and the WHO’s Global Advisory Committee on Vaccine Safety have regularly reviewed the safety of HPV vaccines post-licensure. Recent reviews continue to confirm their safety [20].

Like all vaccines, HPV vaccines can elicit adverse events following immunization (AEFI), ranging from mild to severe [20]. Adverse reactions like syncope, characterized by temporary loss of consciousness, have been noted after HPV vaccination, especially in adolescents. While syncope is seldom life-threatening, it can result in injuries due to falls [21].

Concerns about a potential link between HPV vaccination and autoimmune diseases, such as multiple sclerosis, have been raised [22]. However, a large-scale study of 3,983,824 women, including 789,082 who received the quadrivalent HPV vaccine, found no significant association between the vaccine and multiple sclerosis or other demyelinating disorders, dispelling these concerns [23].

A cohort study from 2012 to 2021 examined national HPV vaccination records and incident diagnostic data for adolescent women aged 9 to 19. Comparing vaccinated and non-vaccinated groups over 180 and 360 day follow-up periods, the study found higher odds ratios for rheumatoid arthritis, juvenile idiopathic arthritis, idiopathic thrombocytopenic purpura, and thyrotoxicosis in the vaccinated group [24]. Nevertheless, several systematic reviews have confirmed that current HPV vaccines are safe and effective, preventing vaccine-type HPV infections and related cellular abnormalities, including pre-cancerous and benign lesions [25].

### 1.2. Global HPV Vaccination Coverage and Progress

Despite the availability of HPV vaccines for over 15 years, global vaccination coverage remains low, significantly exacerbated by the COVID-19 pandemic, which caused declines in coverage, delays in national vaccine program introductions, and increased missed vaccinations, particularly in low- and middle-income countries (LMICs) [26,27]. Cervical cancer incidence and mortality rates vary widely, with countries like Malawi, Zambia, Bolivia, Paraguay, the Maldives, Indonesia, Fiji, and Papua New Guinea reporting the highest rates within their regions [26,27]. Socioeconomic disparities contribute to higher rates in countries with lower Human Development Index (HDI) scores, with Eastern Africa and Eastern Europe experiencing increases [27]. Efforts in North America focus on adolescents through school-based initiatives and primary care integration, yet uptake rates remain low [28]. Latin America faces similar challenges due to limited awareness, safety concerns, cost, and cultural factors [29,30]. In Europe, HPV vaccination is part of all EU/European Economic Area (EEA) national programs, with many adopting gender-neutral approaches [31]. The Middle East shows varied vaccine availability and acceptance [32,33]. At the same time, Asia sees vast differences in coverage, with only half of the Asian National Cancer Centers Alliance (ANCCA) member countries integrating HPV vaccination into national programs [34,35]. From 2010–2019 to 2020–2021, global HPV vaccination coverage among girls dropped from 65% to 50% in LMICs, while high-income countries saw an increase from 61% to 69%, with the COVID-19 pandemic causing significant global coverage delays and approximately 3.8 million girls missing out on vaccination [26,27,28]. Urgent efforts are needed to accelerate global HPV vaccination program implementation and achieve high coverage [26,27,28,29,30,31,32,33,34].

### 1.3. Summary of Challenges in HPV Vaccination: Strategies for Enhancing Vaccination Uptake and Managing Related Issues Based on a Literature Review

#### 1.3.1. Low Awareness of HPV Infection and Vaccination Importance

Many studies have explored the knowledge of HPV and attitudes toward vaccination among various populations. Enhancing health education about HPV vaccination and developing effective strategies to reduce vaccine hesitancy are suggested to significantly improve HPV vaccine uptake, leading to a decrease in the incidence of related diseases and a potential public health benefit (Ran et al., 2022) [35].

Despite the recommendations for the HPV vaccine for adolescent girls throughout Europe, uptake rates vary significantly, necessitating a re-evaluation of HPV education delivery to adolescents across the continent to bridge knowledge gaps [31]. Patel et al. (2016) conducted a systematic review utilizing mixed methods to evaluate HPV and vaccine awareness among European adolescents [36]. Meta-analysis findings indicate that females generally show higher awareness of HPV and its vaccine than males, with factors like age, education level, and vaccination status affecting awareness levels [36]. Nonetheless, a comprehensive understanding of HPV and vaccine efficacy remains limited while uncertainties persist regarding vaccine protection and the necessity of cervical screening post-vaccination [36].

Villanueva et al. (2019) surveyed 536 Spanish nursing students to evaluate their knowledge, attitudes, and intentions regarding HPV and its vaccine [37]. Out of these, 367 (68.4%) students completed the questionnaire. The results revealed moderate knowledge and positive attitudes towards vaccination. Notably, the students’ intention to get vaccinated significantly increased after completing the questionnaire. These findings underscore the importance of educating future nurses about HPV and its vaccine to effectively prevent sexually transmitted diseases [37].

Kornides et al. (2018) investigated the phenomenon of parents reconsidering HPV vaccination for their children after initially refusing it, termed “secondary acceptance” [38]. Their findings indicated that 45% of parents reported secondary acceptance, which was associated with follow-up counselling about HPV vaccination from a healthcare provider. These results underscore the critical role of healthcare providers in encouraging secondary acceptance through consistent and thorough recommendations for HPV vaccination [38]. A summary of challenges in HPV vaccination including strategies for enhancing vaccination rates and managing related issues are reported in Table 1.

#### 1.3.2. The Role of Schools in Bridging the Gap in HPV Vaccination Acceptance and Boosting Coverage

School-based immunization programs (SBIPs) can increase HPV vaccine uptake by including education designed for adolescents. A qualitative study found that parents, recognized as critical decision-makers, emphasized the importance of parent-directed vaccine education within SBIPs [39]. These findings encourage the creation of joint HPV vaccine programs that include the participation of students’ parents to improve SBIPs in British Columbia [39].

Tobaiqy et al. (2023) studied parental understanding, attitudes, and perspectives on HPV in Saudi Arabia, surveying 500 parents [40]. They found that only 11% were aware of HPV’s link to cervical cancer. Most respondents from middle social strata were unfamiliar with the HPV vaccine and hesitant to vaccinate their daughters due to insufficient information and safety concerns. Proposed methods to enhance vaccine acceptance included utilizing social media for campaigns, implementing educational programs in schools and creating a government-organized platform to provide information [40].

To boost vaccination rates, both healthcare professionals and parents need to recognize the importance of vaccinating adolescents before they become sexually active. In a systematic review, Holman et al. (2014) identified critical barriers to HPV vaccination among US adolescents, such as financial constraints, parental attitudes, and the need for more information. Parents often worried about the vaccine’s impact on sexual behavior and perceived low risk of HPV infection. Some parents of sons chose not to vaccinate due to the perceived lack of direct benefits. Healthcare professionals’ recommendations significantly influenced parents’ decisions to give HPV vaccine to their children [41].

Introducing new consent procedures could significantly address the decline in HPV vaccination uptake. Fisher et al. (2022) observed an increase in HPV vaccination rates within a local authority after implementing these new procedures for young women attending school there. However, despite the improvement, disparities among different school types, ethnic groups, and socioeconomic levels persisted [42].

While the school-based HPV vaccination program has effectively reached most young women, issues of accountability and challenges faced by specific groups highlight the need for a multifaceted strategy that addresses cultural and literacy barriers to improve uptake and reduce disparities. Batista Ferrer et al.’s (2016) qualitative study revealed that varying commitment levels among school staff, nurses, parents, and students in returning consent forms hindered access to HPV vaccination. Additionally, beliefs and values related to adolescent sexual behavior, especially within minority ethnic communities, affected vaccine uptake [43].

#### 1.3.3. The Involvement of Healthcare Professionals in HPV Vaccination

Thorough educational campaigns directed towards healthcare professionals (HCPs) and the implementation of effective communication strategies could serve as an efficient approach to encourage vaccine uptake and rectify misconceptions [40,41,42]. Zimet et al. (2013) investigated factors contributing to suboptimal HPV vaccine uptake, such as apprehensions regarding sexual risk compensation and vaccine safety, as well as insufficient recommendations from HCPs. Despite evidence refuting the notion of post-vaccination sexual risk compensation and confirming the safety of HPV vaccines, concerns persist. Notably, inadequate recommendations from HCPs emerged as a significant influencer of non-vaccination [44].

In 2016, Costa et al. conducted a cross-sectional study to identify knowledge gaps in HPV infection and HPV vaccination among medical students. The study revealed significant deficiencies in understanding vaccine indications for HIV-positive individuals and contraindications for pregnant patients. Notably, male students in their early education years showed considerable knowledge gaps. These findings emphasize the need for improved HPV education throughout medical training [45].

Interventions to enhance healthcare providers’ recommendations for HPV vaccination in adolescents are crucial. In a study by Gilkey et al. (2016) involving 1495 parents of 11–17-year-olds in the United States, nearly half reported not receiving any HPV vaccination recommendations from their providers. Among those who did, 16% received low-quality recommendations, and 36% received high-quality recommendations. High-quality recommendations significantly increased vaccine initiation and reduced vaccine hesitancy or postponement [46]. Addressing vaccine disparities among ethnic and racial groups is essential, with improved vaccine knowledge being a key factor. In a systematic review, Kessels et al. (2012) identified several factors associated with higher vaccine uptake: health insurance coverage, older age, prior childhood vaccinations, better vaccine knowledge, increased healthcare utilization, information from healthcare providers, and positive attitudes toward vaccination [12]. In the US, disparities were observed with African American girls having lower rates of vaccination initiation and completion [12].

#### 1.3.4. HPV Vaccination Cost

Lowering or eliminating vaccination costs is crucial for improving accessibility and uptake rates [12,46,47]. Cost-effectiveness evaluations emphasize the public health benefits of HPV vaccination in containing cervical cancer and reducing mortality. However, delivery and program costs hinder vaccine distribution and access [12,46,47]. Zou Z et al. (2020) noted that the cost-effectiveness of HPV vaccination depends on vaccine pricing, highlighting the need for reduced costs to ensure economic feasibility [47]. In their systematic review, Akumbom et al. (2022) examined the cost and cost-effectiveness of HPV vaccination strategies to enhance access. They found that countries without national HPV vaccination programs are developing sustainable methods, such as demonstration programs, to provide vaccines to adolescents [48]. In contrast, countries with established programs focus on cost-effective measures to increase vaccination rates and meet national standards. The finding highlights the need for tailored HPV vaccination programs and intervention studies in low and middle-income countries to improve vaccine access [48].

In the USA, a model assessing the nationwide switch to the nonavalent HPV vaccine found it cost-effective at all coverage levels, despite higher per-dose costs, and beneficial for health outcomes, especially in states with low vaccination rates. Coordinated multi-state policies would maximize health, increase HPV vaccination coverage, and economic benefits (Durham et al., 2016) [49].

#### 1.3.5. Vaccine Delivery Systems and Coverage

Identifying effective delivery methods to improve HPV vaccine coverage is essential. LaMontagne et al. evaluated vaccination coverage in India, Peru, Uganda, and Vietnam, finding high rates (82.6% to 96.1%) due to perceived benefits in cervical cancer protection and disease prevention. Refusals were mainly due to programmatic issues rather than vaccine opposition. The study highlights the importance of diverse delivery strategies and reinforcing favorable motivators to boost vaccine acceptance and uptake [50].

In a review by Smulian et al. (2016), intervention studies focused on increasing HPV vaccination rates, such as parental education and school vaccination requirements, were assessed. The findings showed that diverse, combined strategies effectively boosted vaccination rates, often at a low cost [51]. The most significant improvements were linked to interventions targeting both providers and communities. However, not all interventions significantly increased coverage, underscoring the need for further research to identify the most effective strategies for widespread implementation [51].

Jemal et al. (2013) reported that in 2010, 32.0% of girls aged 13 to 17 in the US received all three doses of the HPV vaccine, with notably lower coverage among the uninsured and in specific Southern states. These results underscore the necessity of bolstering prevention initiatives, particularly for HPV-associated cancers, and elevating vaccination rates [52].

#### 1.3.6. Vaccine Hesitancy

There is limited understanding of how to address parental vaccine hesitancy. While barriers have been identified, prompting factors for vaccine-hesitant parents (VHPs) are underexplored but crucial for effective interventions. Evidence on strategies to improve vaccine uptake among VHPs’ children is scarce, with existing studies using diverse approaches [53].

Gaining insight into obstacles and aids to HPV vaccination is crucial. Zheng et al. (2021), in their systematic investigation, uncovered barriers hindering HPV vaccine uptake, including insufficient understanding of HPV and the vaccine, safety apprehensions, financial limitations, and discrimination related to vaccination [54]. Conversely, factors facilitating vaccination encompassed confidence in vaccine effectiveness and safety, affordability, favorable recommendations, perceived risk of HPV infection, and recognition of vaccine benefits [54,55]. Despite being accessible for 14 years, knowledge deficits endure, underscoring the pressing necessity for educational campaigns aimed at adolescents and young adults to bolster vaccination rates and grasp early vaccination determinants [54].

Beavis et al. (2022) conducted a study aiming to identify potential strategies perceived by vaccine-hesitant parents as motivating for vaccinating their children against HPV. They found that concerns about safety and necessity, frequently impacted by adverse anecdotal reports, were significant factors causing hesitancy [55].

While pediatricians served as the primary source of vaccine information, many parents expressed dissatisfaction with these interactions. Parents indicated a need for comprehensive information regarding vaccine benefits and risks and resources to facilitate discussions with providers. Proposed strategies included detailed discussions with pediatricians, provision of written materials by pediatricians, and facilitation tools to prepare for pediatrician visits [55].

#### 1.3.7. Vaccination Data Monitoring and Surveillance

Strengthening surveillance systems is essential for monitoring HPV vaccination coverage, identifying underserved populations, and tracking vaccine effectiveness and safety. Brewer et al. (2017) assessed the impact of provider training on HPV vaccination recommendations through “announcements” or “conversations” in a randomized trial at 30 clinics across the US. Clinics with announcement-based training saw a 5.4% increase in vaccination coverage among 11- to 12-year-olds compared to control clinics [56]. However, conversation-based training did not lead to significant changes in vaccination coverage. These findings underscore the efficacy of announcement-based training in promoting HPV vaccine initiation among young adolescents [56].

Velentzis et al. (2023) examined whether transitioning Australia’s cervical screening program to HPV testing with genotyping for HPV16/18 could positively follow the impact of the HPV vaccination program started in 2007 on the prevalence of HPV16/18 [57]. The study revealed a significant decrease in HPV16/18 prevalence from 4.85% to 1.67%. Similarly, reductions were observed in oncogenic HPV prevalence from 15.70% to 9.06%. The study revealed that consistent application of a single monitoring method is crucial for accurately assessing changes in HPV prevalence following vaccination [57].

Potter et al. (2015) evaluated linking female records in Michigan’s Immunization Information System (IIS) with the cancer registry [58]. They found that 68% of IIS and 61% of cancer registry records could be matched with birth records, mostly indicating continuous residency. Ultimately, the linkage was achieved for most cases born after 1984. This study showed the feasibility of connecting IIS and cancer registry records, enabling future research into cervical cancer precursors in HPV-vaccinated females [58]. It is clear that there are many and diverse challenges identified in the literature which underscore the need for this systematic review.

This systematic review aims, as required by PRISMA (Supplementary Materials), to be based around PICO (population, intervention, context and outcomes) and to identify the primary challenges (intervention) associated with HPV vaccination (population), propose effective strategies to improve vaccination uptake (intervention) and compile relevant recent evidence (context) to inform policy and practice (outcome) [59].

## 2. Materials and Methods

A systematic review protocol was devised following PRISMA-P and PRISMA guidelines and registered with CRD Prospero (PROSPERO 2024 CRD42024553229) [60]. Searches were conducted in the Web of Science database, which has extensive coverage, using search term combinations based on keywords derived from the literature on HPV vaccination challenges and uptake strategies, as summarized in Table 1.

Keywords included: human papillomavirus, HPV, vaccination, uptake, acceptability, awareness, schools, parents, involvement, healthcare professionals, cost, delivery systems, data monitoring, surveillance, coverage, and hesitancy. The search string, including wildcards (*), was applied to the Web of Science database as follows: human papillomavirus AND (“vaccinat*” OR “increase uptake” OR “acceptab*” OR “aware*” OR “school*” OR “parents” OR “involve*” OR “healthcare professional*” OR “cost” OR “delivery system” OR “data monitor*” OR “surveillance” OR “coverage” OR “hesitancy”).

### 2.1. Eligibility Criteria and Study Selection

Peer-reviewed articles in English, published from 1 January 2020, to 1 May 2024, were included. The review focused on evidence on HPV vaccination challenges and uptake strategies from randomized controlled trials (RCTs), non-RCTs, and observational, qualitative, and cross-sectional studies. Review articles, case reports, study protocols, editorials and commentaries were excluded. Titles, abstracts, and full texts were obtained and screened. Inclusions and exclusions were recorded following PRISMA Statement guidelines [61].

A data extraction tool was developed to capture the following: focus of interest, types of studies, geographical locations where the research was conducted, specific mentions of HPV vaccination uptake, strategies to improve vaccine accessibility, key findings, and areas for further research.

### 2.2. Screening, Extracting and Data Synthesis

After duplicate studies were removed, titles were screened by first author to exclude clearly irrelevant studies. In the second round, abstracts and keywords were independently reviewed by both authors to further refine the selection noting agreed exclusions. Subsequently, a thorough manual review of the full text articles was conducted by both authors to assess relevance and quality. This rigorous manual screening process ensured a comprehensive and accurate review.

Raw data were extracted and categorized as qualitative data fragments within an existing taxonomy. Key findings were summarized in themes describing strategies identified to enhance HPV vaccination uptake. Due to the qualitative nature of this study, no conversions of effect sizes or complex aggregations were performed, so this review focuses on simple descriptive analysis. This approach will ensure the data is organized and aligned with the variables of interest listed in the review protocol methods section, providing clear strategies for improving HPV vaccination uptake.

### 2.3. Quality and Risk of Bias Assessment

The studies were evaluated by the first author using the National Institutes of Health (NIH) study quality assessment tool designed for observational cohort and cross-sectional studies and randomized clinical trials [62].

### 2.4. Statistical Analysis

A table was created to present key findings and strategies for increasing HPV vaccine uptake while descriptive statistics summarized the included and excluded studies according to PRISMA guidelines [61].

## 3. Results

**Figure 1:**
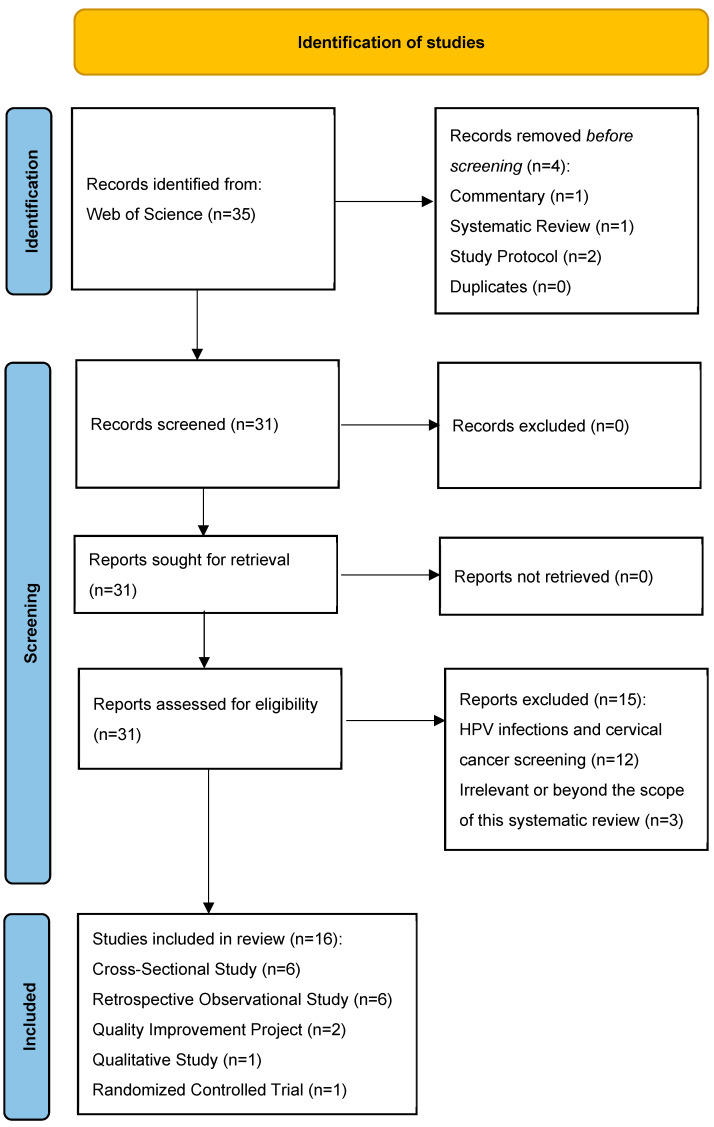
PRISMA flow diagram reporting search results [61].

Searches conducted in the Web of Science database, using identified keywords, limited to 1 January 2020 and 1 May 2024, yielded 35 articles. Inclusions and exclusions are detailed in the PRISMA Statement Flow Diagram in Figure 1 [61].

Four articles were excluded, as they consisted of a commentary (n = 1), a systematic review (n = 1) and study protocols (n = 2). The remaining 31 articles were further screened. These included cross-sectional studies (n = 13), retrospective observational studies (n = 8), quality improvement projects (n = 2), qualitative studies (n = 6), and randomized controlled trials (n = 2). Fifteen articles were excluded for focusing on HPV infections and cervical cancer screening (n = 12) or being irrelevant (n = 3). Sixteen articles were included: cross-sectional studies (n = 6), retrospective observational studies (n = 6), quality improvement projects (n = 2), a qualitative study (n = 1) and a randomized controlled trial (n = 1) which are summarized in Table 2.

Five key strategies were identified with the potential to improve HPV vaccination uptake:

(a) The importance of the parental role and school engagement to enhance HPV vaccination uptake, supported by five articles: Aruho et al., 2022, Rockliffe et al., 2020; Elenwo et al., 2023; de Fouw et al., 2023 and Sisnowski et al., 2021 [63,64,65,66,67].
(b) The use of technology and multimedia educational tools to enhance HPV uptake, supported by five articles: Meadows et al., 2024; Pfingstag, 2024; Panagides et al., 2023; Huang et al., 2022 and Berenson et al., 2020 [68,69,70,71,72].
(c) The role of healthcare providers, especially sexual health clinicians, in discussing HPV vaccination and enhancing accessibility, supported by three articles: Grewal et al., 2021; Armstrong et al., 2023 and Russell et al., 2020 [73,74,75].
(d) Implementing multicomponent and systems-based interventions to increase HPV vaccination uptake, supported by two articles: Dang et al., 2023 and Davies et al., 2023 [76,77].
(e) Targeted interventions to improve vaccination uptake among specific immigrant groups, supported by one article: Hertzum-Larsen et al., 2020 [78].

**Table 2:** HPV vaccination challenges and strategies to enhance uptake: included studies (n = 16).

Type of Study and Geographical Location	Key Findings	Strategy to Increase HPV Uptake	Reference
Cross-sectional study, Uganda	Low HPV vaccination coverage among adolescents in Gulu Municipality, which is influenced by parental perceptions and marital status.	Efforts to improve vaccination rates should target the parents of adolescents.	Aruho et al. (2022) [63]
Retrospective observational study, Denmark	PV vaccination rates differed across countries and regions. Immigrants generally had lower vaccination rates compared to native individuals.	Targeted interventions are needed to improve uptake among specific groups of immigrants and their descendants.	Hertzum-Larsen et al. (2020) [78].
Retrospective observational study Canada	Few men living with HIV have received the HPV vaccine, likely due to low awareness, high costs, and lack of physician recommendations.	Primary care and HIV clinics could be key in increasing vaccination rate.	Grewal et al. (2021) [73]
Quality improvement project, USA	The implementation of a multicomponent, systems-based intervention resulted in an increase in catch-up HPV vaccination rates at a sexual and reproductive health clinic.	The intervention included electronic health record prompts, in-clinic education, and scheduling the next visit during the current one.	Pfingstag (2024) [69]
Retrospective observational study, USA	Implementation of HPV CHAT, a provider education intervention focused on communication strategies for recommending HPV vaccination, in seven family medicine and pediatric clinics in the USA, resulted in minimal effect on increasing the HPV vaccination rate.	New strategies are required to overcome provider barriers to HPV vaccination.	Meadows et al. (2024) [68]
Quality improvement project, USA	The multilevel intervention significantly increased HPV vaccination initiation and completion rates among adolescent patients aged 11–17 at a rural health clinic.	Incorporating tailored HPV vaccination reminder postcards, clinic-wide training, quarterly data reviews, examination room posters, and educational handouts.	Dang et al. (2023) [76]
Retrospective observational study, Australia	The return of consent forms is a significant logistical challenge to HPV vaccine uptake. Implementing a comprehensive intervention with logistical elements can improve consent form returns, though it may not significantly boost vaccine uptake.	Effective stakeholder collaboration and addressing multiple levels of influence to optimize school-based vaccination efforts.	Davies et al. (2023) [77]
Cross-sectional study, South Africa	Most young women lack access to the national HPV vaccine program.	Participants would accept the vaccine if it were free and recommended by a healthcare professional.	Russell et al. (2020) [75]
Qualitative study, UK	Barriers to delivering HPV vaccination in schools included limited resources, fear of the vaccination, and poor return of consent forms.	Optimal HPV vaccination delivery relies on school engagement and adequate time for vaccination teams to promote uptake.	Rockliffe et al. (2020) [64]
Cross-sectional study, USA	Improvements were observed in both knowledge and vaccination after the intervention of educational films on HPV vaccination intent in urban and rural.	Video interventions can promote HPV vaccination in rural areas and can be integrated into school health education programs and distributed to rural physicians and parent.	Panagides et al. (2023) [70]
Randomized controlled trial, China	Sharing information about a sexually transmitted infection raised the willingness to vaccinate a 6-year-old son and a 6- or 12-year-old daughter.	Although this study showed that messaging had a limited effect on willingness to vaccinate against HPV, additional research is necessary to improve HPV vaccine uptake when it is not publicly funded.	Huang et al. (2022) [71]
Cross-sectional study, UK	The most common reason for not getting vaccinated was the belief that it was unnecessary and not recommended by a healthcare provider.	Sexual health clinicians should actively discuss HPV vaccination and enhance appointment accessibility and reminders to improve vaccination uptake and completion rates.	Armstrong et al. (2023) [74]
Cross-sectional study, USA	Maternal education was the strongest predictor of teen HPV vaccination. Mothers with less education were more likely to intend vaccination for teens not yet vaccinated than college-educated mothers.	Understanding maternal characteristics can help develop targeted strategies to improve vaccine uptake.	Elenwo et al. (2023) [65]
Cross-sectional study, Uganda	Involving men and their communities can enhance the acceptance and uptake of prevention services. Focus group discussions with 67 men in Western Uganda revealed support for cervical cancer prevention while highlighted gaps in understanding.	To improve the uptake of screening and HPV vaccination, educating men and actively involving them in awareness programs is essential.	de Fouw et al. (2023) [66]
Retrospective observational study, USA	A patient navigator (PN) program in pediatric clinics significantly increased HPV vaccination rates among 9–12 year olds by providing information and scheduling follow-up doses.	To boost HPV vaccine uptake, implementing on-site PN programs in clinics is recommended, especially for younger patients.	Berenson et al. (2020) [72]
Retrospective observational study, Australia	School and population characteristics are associated with low HPV vaccination initiation and completion. Factors strongly correlated with low initiation included small school size, location and special educational needs.	These findings will guide further research and help target initiatives to improve vaccination uptake in schools with lower coverage profiles.	Sisnowski et al. (2021) [67]

## 4. Discussion

These strategies, backed by multiple studies, involved an extensive literature review, highlighted the multifaceted approach required to improve vaccination rates, providing a solid foundation for policy and stakeholder actions. This study contributes to the literature by synthesizing diverse strategies for enhancing HPV vaccination uptake and underscores the necessity for multifactorial interventions. Policymakers and stakeholders, working together, can leverage these findings to develop comprehensive programs involving schools, parents, technology and healthcare providers to boost vaccination rates. Training programs for healthcare providers and targeted interventions for under-represented groups, can further augment these efforts. By promoting multicomponent and systems-based interventions, this study informs policy and stakeholder actions, fostering a sense of teamwork aimed at significantly improving public health outcomes related to HPV morbidity and mortality.

In regions with lower socioeconomic status worldwide, extensive efforts should be directed towards HPV vaccination [79]. These efforts encompass WHO initiatives to surmount barriers to access, enhance vaccine coverage, and augment public awareness [16]. Organizations like the global alliance for vaccines and immunization (GAVI) have played pivotal roles in supporting HPV vaccines uptake in low-income countries. Moreover, there is a growing emphasis on integrating HPV vaccination into existing healthcare services, including routine immunization programs and school-based health initiatives, to effectively reach marginalized populations [80]. Additionally, ongoing research uptake strives to formulate cost-effective vaccination strategies, address vaccine hesitancy, and fortify healthcare infrastructure, ensuring sustainable HPV vaccination campaigns in these regions [81].

While this systematic review did not investigate psychological and behavioral theories and messaging strategies for increasing HPV vaccination uptake, as two other systematic reviews have been conducted to assess messaging strategies and the population-level impacts of HPV vaccination [82,83].

The first review examined how messaging about HPV vaccination for preventing sexually transmitted infections, anogenital warts (AGW) and cancer influences vaccination intentions and initiation. It identified 25 studies using various methods to compare messages, including randomized trials, surveys, and qualitative approaches. The results indicated mixed support for cancer prevention messages with some studies finding equal or more significant support for messages focusing on STI/AGW prevention. The review highlighted significant variability in messaging effectiveness across different populations, emphasizing the need for a deeper understanding of HPV’s myriad health outcomes to enhance vaccination uptake [82].

The second review and meta-analysis updated evidence on the population-level impact of HPV vaccination on HPV infections, AGW diagnoses, and grade 2+ CIN, analyzing data from 60 million individuals across 65 studies; the findings showed significant reductions in HPV 16 and 18 infections, AGW diagnoses, and CIN2+ among vaccinated cohorts. The study underscored the more significant and faster impact of vaccination programs with multiple age cohorts and high coverage. The results strongly support the effectiveness of HPV vaccination in preventing cervical cancer and endorse the WHO recommendation for vaccinating multiple age cohorts of girls. This highlights promising signs for the potential elimination of cervical cancer with sufficient vaccination coverage [83].

Of note, interventions to enhance HPV vaccination uptake should be grounded in robust theoretical models. A review of 34 studies on HPV vaccination behaviors found that 31 studies employed psychological health behavior models, and three utilized sociocultural theories, with outcomes measured across professionals, parents, and young women. Despite this, only half of the quantitative studies reported goodness-of-fit and variability data, complicating the assessment of the theoretical contributions [84].

Assessing health belief methods (HBM) and theory of planned behavior (TPB) through a survey revealed that TPB consistently outperformed HBM, with key predictors including subjective norms, self-efficacy, and vaccine cost [85]. An observational study of undergraduates (n = 190; 66.8% female) showed that social norms from parents, friends, and doctors indirectly influenced vaccine intentions via self-efficacy and attitudes. Enhancing self-efficacy and attitudes through positive communication with healthcare providers, and significant others, is crucial in emphasizing the role of social support in promoting HPV vaccination [86].

### Strengths and Limitations

This review synthesizes the current state of HPV vaccination strategies, presenting a clear picture of past efforts, progress, challenges and future directions. By addressing the identified challenges and leveraging proposed strategies, there is potential to significantly improve HPV vaccination coverage and, subsequently, public health outcomes.

This study’s strengths include extensive referencing, comprehensive scope, diverse evidence base, thematic categorization, focus on recent data and a holistic approach considering multiple perspectives. While this systematic review is thorough throughout with extensive literature included, starting in the Introduction section, it is essential to note that it was conducted using one database, which may introduce potential biases. However, that literature included a robust background, discussing key and influential studies sourced from various databases over many years.

Furthermore, the validity of screening and extracting processes was assessed independently by two reviewers sufficient to offer an introduction for any readers new to the research area. This systematic review consolidated the evidence offered in the Introduction section by gathering recent articles based on keywords, also from the literature, and utilizing a robust reviewer-based methodology to manage potential review workload [61].

## 5. Conclusions

Based on the study findings, future research should focus on increasing awareness about HPV infection and vaccination founded on the extensive, evidence-based literature included in this systematic review. It should involve schools and parents to bridge the vaccination gap, enhancing acceptance and coverage through targeted interventions, actively involving healthcare professionals, addressing vaccination costs, improving vaccine delivery systems and global coverage while combating vaccine hesitancy. Additionally, utilizing technology and social media for these tasks can be pivotal. These steps are essential for optimizing HPV vaccination uptake and ensuring broad public health benefits and outcomes.

The review highlights critical challenges in HPV vaccination and identifies areas which need targeted interventions. The data highlights the importance of coordinated efforts across all sectors, including schools, healthcare professionals and policymakers, again, to enhance vaccination coverage and uptake. Future research should focus on overcoming these identified barriers and implementing effective strategies to improve HPV vaccine uptake. Implementing evidence-based interventions and fostering global cooperation can help public health agencies strive towards achieving higher HPV vaccine coverage rates alleviating the burden of HPV-related morbidity and mortality.

## Figures and Tables

**Table 1:** A summary of challenges in HPV vaccination: strategies for enhancing vaccination rates and managing related issues.

Challenges	Recommended Strategies for Enhancing HPV Vaccination Uptake	Description	References
Low awareness of HPV infection and vaccination importance	Run awareness campaigns Improve health education and empower healthcare providers Target education Provide counseling and support to parents	* Run communication campaigns and provider training to mitigate concerns and build trust in HPV vaccines * Develop targeted programs to address knowledge gaps and misconceptions about HPV vaccination * Tailor HPV education delivery to adolescents, considering demographic variations in awareness levels, to ensure relevance and accessibility * Equip providers with knowledge and resources to counsel patients and parents effectively, encouraging proactive discussions and follow-up counselling * Foster secondary acceptance: provide follow-up counselling and support to parents who initially declined vaccination	Ran et al. (2022) [35] Patel et al. (2016) [36] Villanueva et al. (2019) [37] Kornides et al. (2018) [38]
The role of schools and parents in bridging the gap in HPV vaccination acceptance and boosting coverage	Incorporate tailored education in school-based immunization programs (SBIPs) Recognize parents as key decision-makers Raise parental awareness Address concerns Implement consent Address accountability issues	* Stress the importance of parent-directed vaccine education within SBIPs * Develop stronger collaborative HPV vaccine curricula involving both students and parents to enhance SBIPs and increase vaccination rates * Raise awareness among parents about the link between HPV and cervical cancer through educational campaigns and sessions in schools * Address concerns about HPV vaccine safety and efficacy through social media awareness campaigns and government-led platforms advocating for vaccination * Address accountability issues and obstacles specific groups face through a comprehensive, multi-faceted approach	Brohman et al. (2024) [39] Tobaiqy, et al. (2023) [40] Holman et al. (2014) [41] Fisher et al. (2022) [42] Batista Ferrer et al. (2016) [43]
The involvement of healthcare professionals in HPV vaccination	Enhance healthcare provider education	* Conduct thorough educational campaigns targeting healthcare professionals (HCPs) to address misconceptions and encourage vaccine uptake * Enhance education regarding HPV and its vaccine throughout medical training for medical students to bridge knowledge gaps * Implement interventions to improve the quality of healthcare providers’ recommendations for HPV vaccination among adolescents * Address disparities in vaccine receipt among ethnic and racial groups by providing accurate information from reliable sources and increasing healthcare utilization	Zimet et al. (2013) [44] Costa et al. (2020) [45] Gilkey et al. (2016) [46] Kessels et al. (2012) [12]
HPV vaccination cost	Reduce cost barriers	* Reduce the cost of vaccination or provide it for free to enhance accessibility and uptake rates * Prioritize identifying cost-effective interventions to elevate vaccination rates and align with nationally recommended standards in countries with established national vaccination programs * Coordinated multi-state policies would maximize health, increase HPV vaccination coverage, and economic benefits	Zou et al. (2020) [47] Akumbom et al. (2022) [48] Durham et al. (2016) [49]
Vaccine Delivery Systems and coverage	Strengthen vaccine delivery systems	* Implement diverse delivery methods tailored to different settings and populations * Reinforce positive motivators for vaccine acceptance, emphasizing perceived cervical cancer protection and disease prevention benefits * Employ combined intervention strategies, such as educational requirements for parents and school-based HPV vaccination programs, to enhance coverage * Focus on improving vaccination rates among underserved populations, including the uninsured and residents of specific geographic regions	LaMontagne et al. (2011) [50] Smulian et al. (2016) [51] Jemal et al. (2013) [52]
Vaccine Hesitancy	Address vaccine hesitancy	* Explore motivating factors for vaccine-hesitant parents (VHPs) to develop effective interventions * Address barriers hindering HPV vaccination uptake, such as safety concerns and financial limitations * Promote confidence in vaccine effectiveness and safety, affordability, and favorable recommendations * Provide comprehensive information regarding vaccine benefits and risks to address safety and necessity concerns * Improve interactions with pediatricians by facilitating detailed discussions and providing written materials	Williams (2014) [53] Zheng et al. (2021) [54] Beavis et al. (2022) [55]
Vaccination Data Monitoring and Surveillance	Enhance data monitoring and surveillance	* Implementing robust surveillance systems is essential for monitoring HPV vaccination coverage rates and tracking vaccine effectiveness and safety over time * Transitioning cervical screening programs to HPV testing with genotyping, as done in Australia, can effectively monitor the impact of HPV vaccination programs on HPV prevalence * Connecting records between Immunization Information Systems (IIS) and cancer registries * Facilitates monitoring of cervical cancer precursors in HPV immunization-eligible females	Brewer et al. (2017) [56] Velentzis et al. (2023) [57] Potter et al. (2015) [58]

## Supplementary Materials

The following supporting information can be downloaded at: https://www.mdpi.com/article/10.3390/vaccines12070746/s1, Table S1: PRISMA checklist.
